# Quantitative analysis of {332}〈113〉 twinning in a Ti-15Mo alloy by *in situ* scanning electron microscopy

**DOI:** 10.1080/14686996.2018.1475824

**Published:** 2018-06-07

**Authors:** Ivan Gutierrez-Urrutia, Cheng-Lin Li, Xin Ji, Satoshi Emura, Koichi Tsuchiya

**Affiliations:** a National Institute for Materials Science, Tsukuba, Japan; b Graduate School of Pure and Applied Sciences, University of Tsukuba, Ibaraki, Japan

**Keywords:** Twinning, bcc-Ti alloys, *in situ* SEM, Microstructure, Quantitative characterization, 10 Engineering and Structural materials, 106 Metallic materials, 503 TEM, STEM, SEM

## Abstract

We have performed quantitative analysis of {332}〈113〉 twinning in a β-Ti-15Mo (wt.%) alloy by *in situ* scanning electron microscopy and electron backscattering diffraction (EBSD). Microstructure-twinning relations were evaluated by statistical analysis of the evolving twin structure upon deformation at room temperature. Our analysis reveals that at the early stages of deformation (*ε* < 1.5 to 2.0%), primary twinning is mainly determined by the applied macroscopic stress resolved on the twin system. Most of the primary twins (~70–80% of the analyzed twins) follow Schmid’s law with respect to the macroscopic stress, and most of the growth twins (~ 85% of the analyzed twins) correspond to the higher stressed variant. In the grain size range studied here (40–120 μm), we find that several twin parameters such as number of twins per grain and number of twins per grain boundary area exhibit grain size dependence. We ascribe these effects to the grain size dependence of twin nucleation stress and apparent critical resolved shear stress for twinning, respectively.

## Graphical abstract

**Figure:**
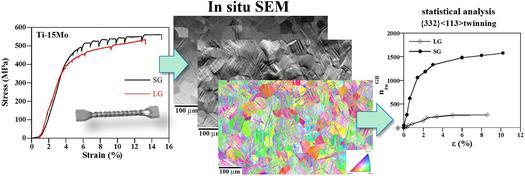

## 1. Introduction

Deformation twinning involves the cooperative motion of a large number of partial dislocations on periodic and adjacent spaced twinning planes. The crystallographic twinning laws specify the twinning plane and the direction of shear. In bcc metals, {112}〈111〉 twinning is the common twinning system, which involves the cooperative gliding of 1/6 〈111〉 twin dislocations [1,2]. However, in bcc β-Ti alloys the operative twinning system is {332}〈113〉[3–9]. Tobe et al. suggested that the activation of this twinning mode is associated to the low shear modulus along 〈110〉 directions on {110} planes (*G*_110_ = ((*c*_11_−*c*_12_)/2)) of β phase, which favors the specific atomic shuffling involved in the {332}〈113〉 twinning system [3]. According to the crystallographic theory of twinning by Bilby and Crocker [10], {332}〈113〉 twinning involves homogeneous shear on {332} twinning planes with shear magnitude of 0.3536 coupled with atomic shuffling on {011} planes along 〈011〉 directions. Twinning dislocations are required to accomplish the shear deformation and they can only glide in the twinning plane so that the lattice correspondence is not disrupted. Experimental data in cubic metals indicate that twinning occurs on the most highly stressed twin planes and hence, a critical resolved shear stress for twinning has been commonly defined [1,11]. Twins have been observed mostly to occur in grains whose orientation results in a high resolved shear stress on the twin system [1,12]. In particular, several works in β-Ti alloys have reported that {332}〈113〉 twinning follow Schmid’s law with respect to the macroscopic applied stress [7,13,14]. However, recent reports on statistical analysis of deformation twinning have shown that this process can be also activated in significant number of grains (~40 to 60% in hcp metals) that are not favorably oriented for twinning [15–18], which makes the validity of the Schmid’s law questionable. Furthermore, in polycrystals twinning strongly depends on microstructure features such as grain size, grain boundaries, pile-ups, crystal defects, slip bands, twin interfaces and cracks [1,12,19–25]. For instance, grain boundaries (including triple lines and quadruple points) are the primary locations for stress concentrations in polycrystals that can supply the energy necessary to overcome activation barriers for twin nucleation to occur. It is thus clear that these microstructure features play a key role in the nucleation and propagation behavior of twins in a polycrystal. In the {332}〈113〉 twinning system several studies have addressed the role of crystallographic grain orientation [7,13,14] and grain size [13]. However, these relations have not been statistically investigated so far.

The present work aims at investigating microstructure-twinning relations in the {332}〈113〉 twin system in a β-Ti alloy by statistical microstructure analysis. The evolution of primary twin structure upon deformation in a polycrystalline β-Ti model alloy, namely, Ti-15Mo (wt.%), was evaluated by *in situ* scanning electron microscopy (SEM) at several strain levels up to *ε* ~ 10%. Statistically significant microstructure area was evaluated by SEM and electron backscattering diffraction (EBSD). The roles of crystallographic grain orientation, grain size and resolved applied stress on twin variant and number of twins per grain and per grain boundary area were investigated in detail.

## 2. Experimental section

### 2.1. Experimental procedure

The Ti-15Mo (wt.%) alloy was prepared by cold crucible levitation melting under Ar gas atmosphere. The ingot was hot forged at 1000 °C to 40% thickness reduction and thereafter hot rolled at 900 °C to 75% thickness reduction followed by air-cooling. The hot-rolled material was subsequently solution-treated for 1 h at 900 °C followed by water quenching. Thereafter, it was annealed for 1 h at annealing temperatures that ranged between 780 and 1000 °C to generate microstructures with different grain sizes. We selected two annealing conditions, namely 780 and 1000 °C, which resulted in annealed microstructures with average grain sizes of 40 μm (referred to as SG) and 120 μm (referred to as LG), respectively. Flat dog-bone tensile samples with gage dimensions of 2.0 mm wide, 1.0 mm thick and 36 mm long were machined out of the annealed material. The samples were machined with the tensile axis parallel to the rolling direction (RD). *In situ* SEM tensile tests were conducted in a tensile stage manufactured by TSL Solutions Japan (Sagamihara-shi, Japan) using a 1000 N load cell. Mechanical tests were performed at room temperature at a constant displacement rate of 2 × 10^−4^ s^−1^ up to strain of *ε* ~ 10%. We used a screw-driven tensile stage placed inside a Sigma Zeiss field emission gun scanning electron microscope (FEG-SEM). The crosshead displacement of the mechanical testing machine was continuously recorded during the test. The strain values were estimated from the displacement measurements taking into account the gage length of the samples. Backscattered electron (BSE) images were taken before loading and during interruptions throughout the tensile test. The investigated area was about 2.5 × 4.0 mm^2^. About 10 strain levels per tensile test were analyzed. SEM images were taken in a Sigma Zeiss field emission gun scanning electron microscope (FEG-SEM) equipped with a TSL orientation imaging microscopy (OIM) EBSD system. EBSD maps were performed at 20 kV with a step size ranging between 0.5 and 5 μm.

### 2.2. Statistical twin analysis procedure

**Figure 1.**
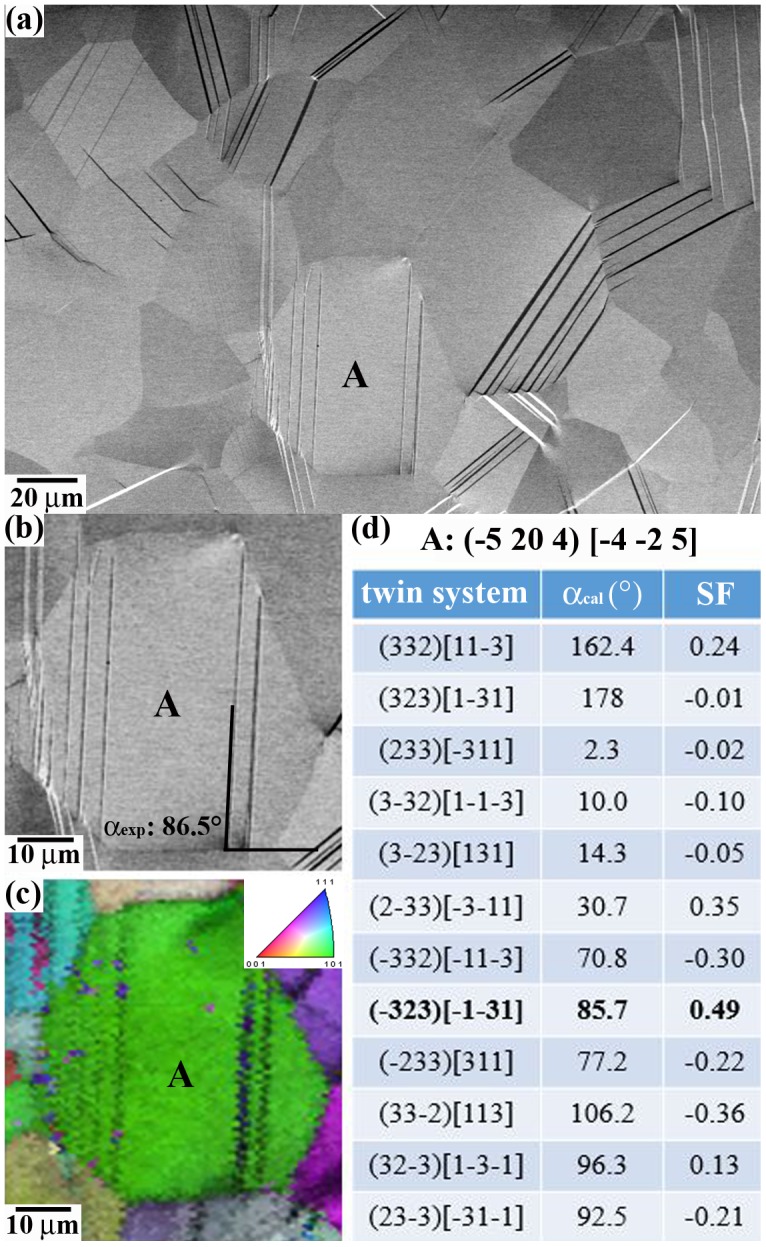
Scheme of the twin plane trace approach. (a): BSE-SEM image of the twin structure of the SG sample strained to 425 MPa/*ε*: 0.3%. (b)–(d): Example of twin plane analysis of grain A marked in (a). *α*_exp_: angle between surface twin plane trace and rolling direction; *α*_cal_: angle between rolling direction and trace projection of the twin plane onto the RD-TD plane; SF: Schmid factor.

The present analysis of twin nucleation and propagation phenomena is based on a 2D SEM characterization approach of surface grains. In our analysis, we have only considered twins that significantly contribute to the overall plastic deformation, i.e. micron-scale twins that are associated with a nucleation event followed by in-grain propagation of a detectable twin. It is also worth commenting that the present 2D surface analysis of twinning may differ to that of the subsurface due to the smaller mechanical constraint associated to the free surface. This effect may lead to different intergranular compatibility strain/stresses in surface grains than those occurring in subsurface grains. As twin nucleation is a stress-assisted process, the nucleation of twins at grain boundaries may be enhanced in some adjacent surface grains and unfavored in others. In order to minimize these effects and obtain sound analysis of twinning, we have performed a large statistical analysis of surface twinning events (about 350 events per sample were evaluated). Active twin systems were identified by the twin place trace approach depicted in Figure 1. The approach consists in the calculation of the best matching between the experimental angle *α*_exp_ (twin plane trace, rolling direction) determined from SEM-BSE images (Figure 1(a) and (b)), and calculated angles *α*_cal_ (rolling direction, trace projection of the twin plane onto the RD-TD plane) of the 12 {332}〈113〉 twin systems of a crystal grain (Figure 1(d)). Crystal grain orientation was determined by EBSD (Figure 1(c)). Schmid factors of active twin variants were calculated with respect to the sense and direction of the macroscopic loading, using the twin plane (*K*_1_) and shear direction (*η*_1_). For each grain, the twelve possible twin variants *v*(*i*) were classified in order of decreasing Schmid factor *m*(*i*) (i: 1, … 12). Variant *v*(1) has the highest Schmid factor *m*(1) and the twelfth variant *v*(12) the lowest *m*(12).

## 3. Results

### 3.1. Twin structure

**Figure 2.**
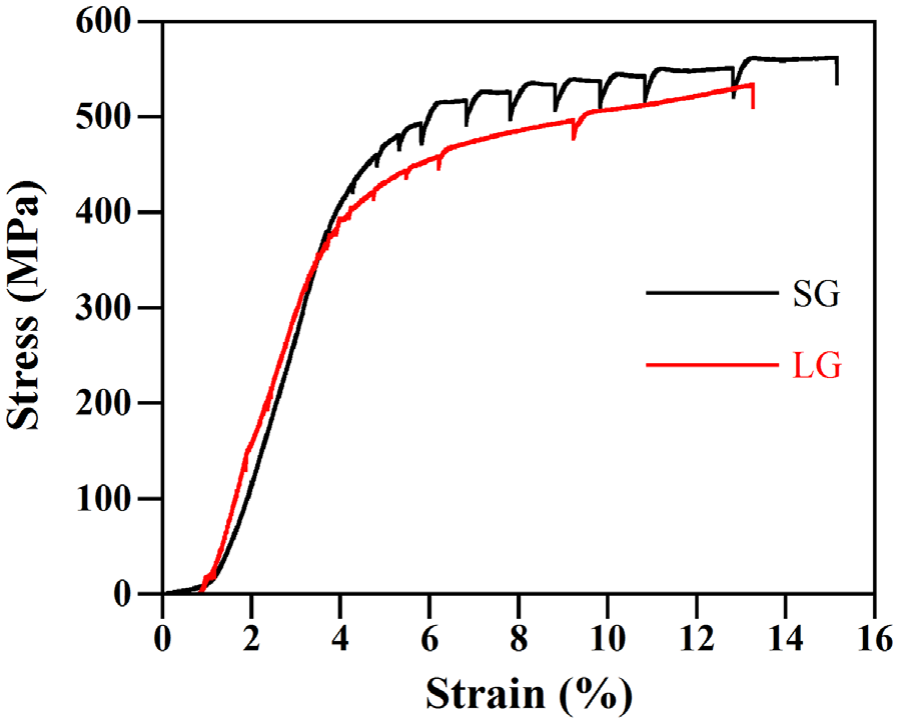
Stress-strain curves of the tensile strained samples.

Notes: The load drops indicate the stress relaxation that occurred when the experiment was paused and BSE-SEM images were taken. SG: Fine-grained sample; LG: Coarse-grained sample.

**Figure 3.**
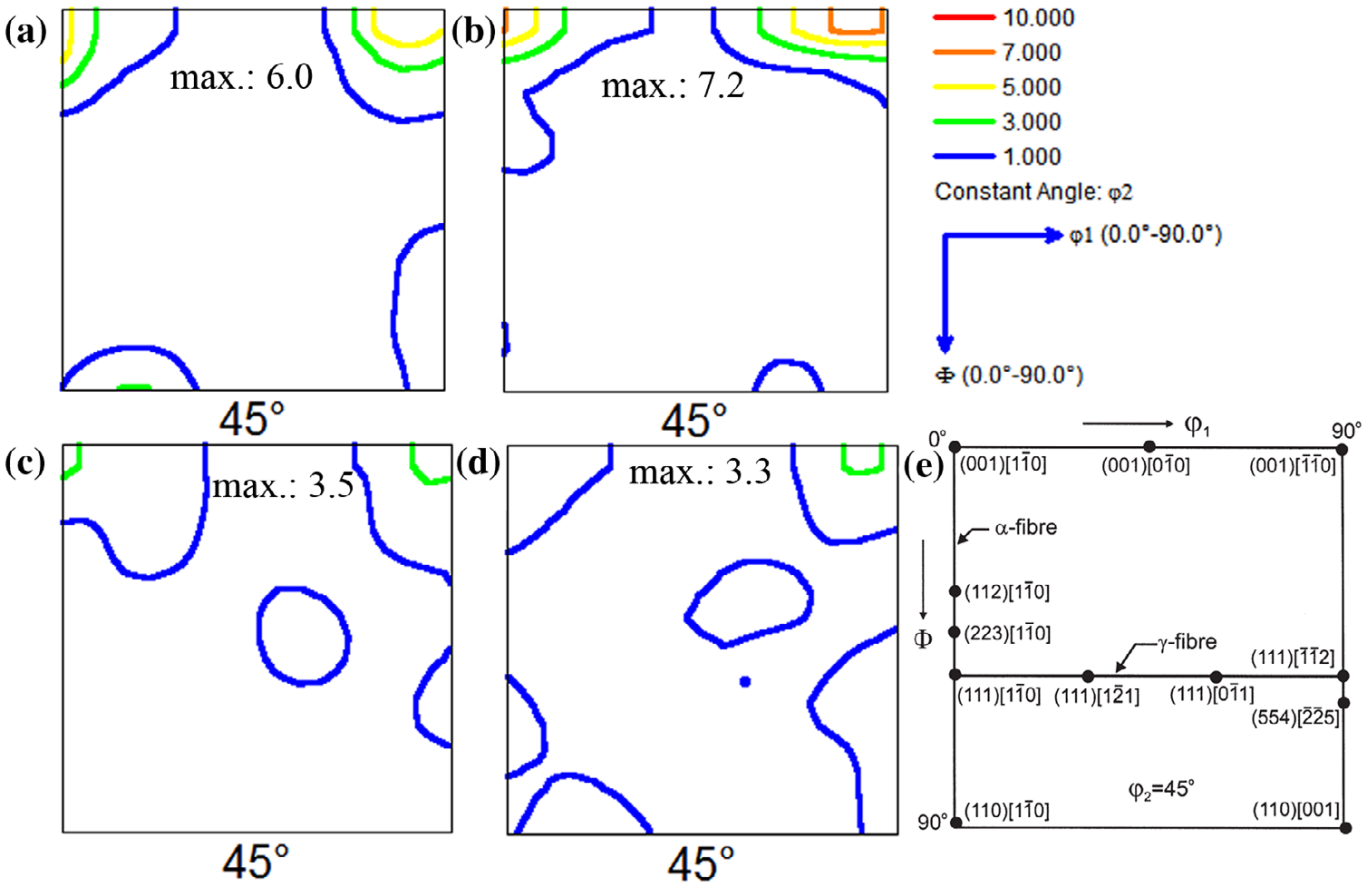
*ϕ*_2_ = 45° ODF sections. (a) Annealed SG sample; (b) Annealed LG sample; (c) SG sample, *ε*: 8.1%; (d) LG sample, *ε*: 8.8%. (e) Drawing of the most relevant texture orientations in bcc metals located in the *ϕ*_2_ = 45° ODF section.

The tensile curves of the interrupted tests for the small grain (SG) and large grain (LG) samples of Ti-15Mo (wt.%) are shown in Figure 2. The load drops indicate the stress relaxation that occurred when the experiment was paused and BSE-SEM images were taken. The yield strength (YS) of the SG sample was 425 MPa, which is 1.12 times greater than that of the LG sample (370 MPa). These YS values are typical of Ti-15Mo alloys with micrograin size [26,27]. The macrotexture of annealed materials mainly consists of the {001}〈110〉 texture component, which is typical of recrystallized β-Ti alloys [28–30], Figures 3(a) and (b). This texture component belongs to the α-fiber (〈110〉 parallel to the rolling direction. Upon tensile straining up to *ε* ~ 10%, the deformation texture is similar to that of the annealed state, Figures 3(c) and (d). These figures show the *ϕ*_2_ = 45° orientation distribution function (ODF) section of the SG and LG samples strained to *ε* = 8.1 and 8.8%, respectively. It can be seen that the texture is formed by the {001}〈110〉 texture component with lower intensity than that in the annealed state and a small {111}[31] texture component, which is commonly observed in highly deformed β-Ti alloys [28,30]. This texture component is associated to 〈111〉 slip [32].

**Figure 4.**
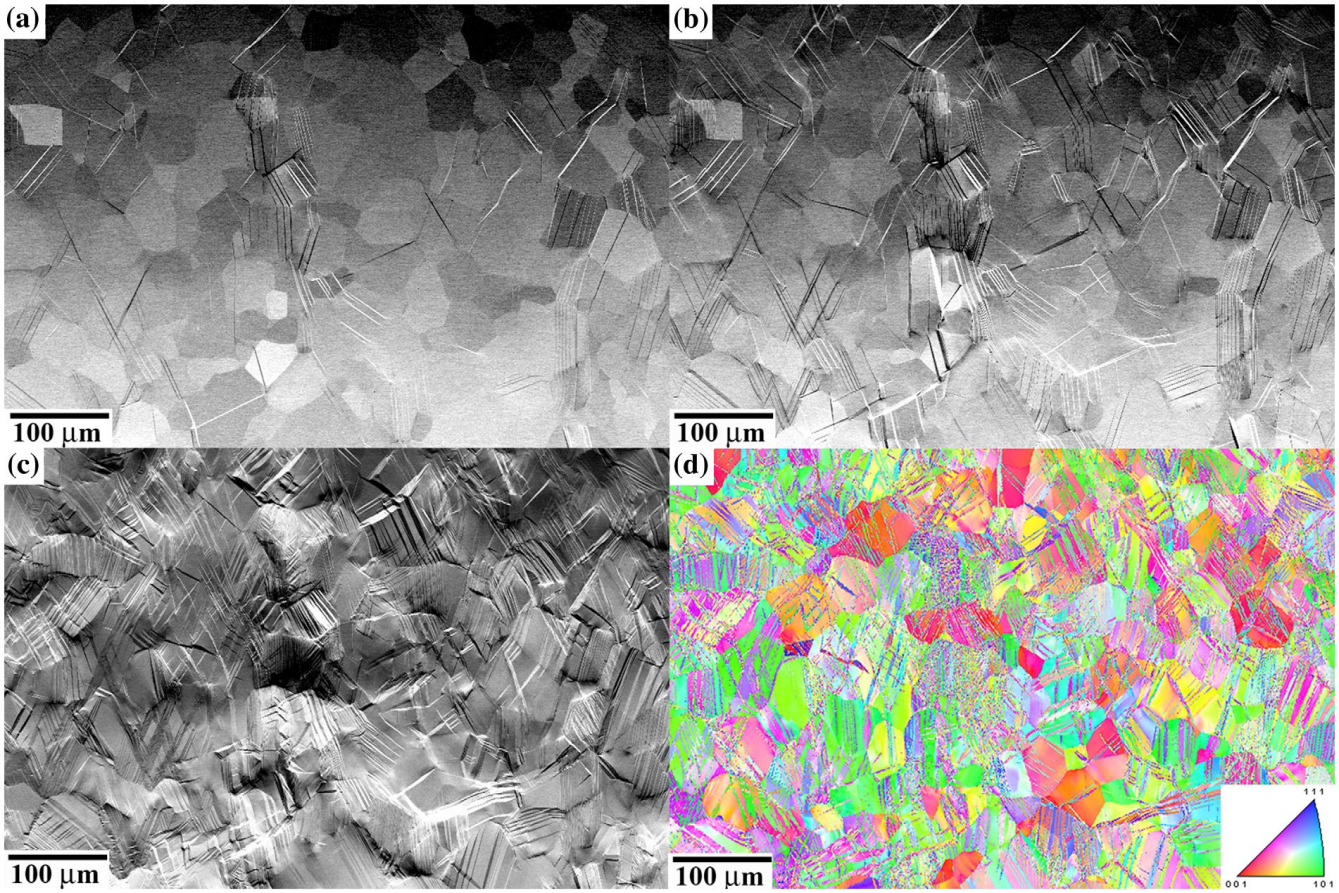
BSE-SEM images of the evolution of {332}〈113〉 twin structure upon deformation in the SG sample. (a) 425 MPa/*ε*: 0.3%; (b) 495 MPa/*ε*: 1.4%; (c) 560 MPa/*ε*: 8.1%. (d) EBSD map along tensile direction. Sample deformed to 495 MPa/*ε*: 1.4%.

**Figure 5.**
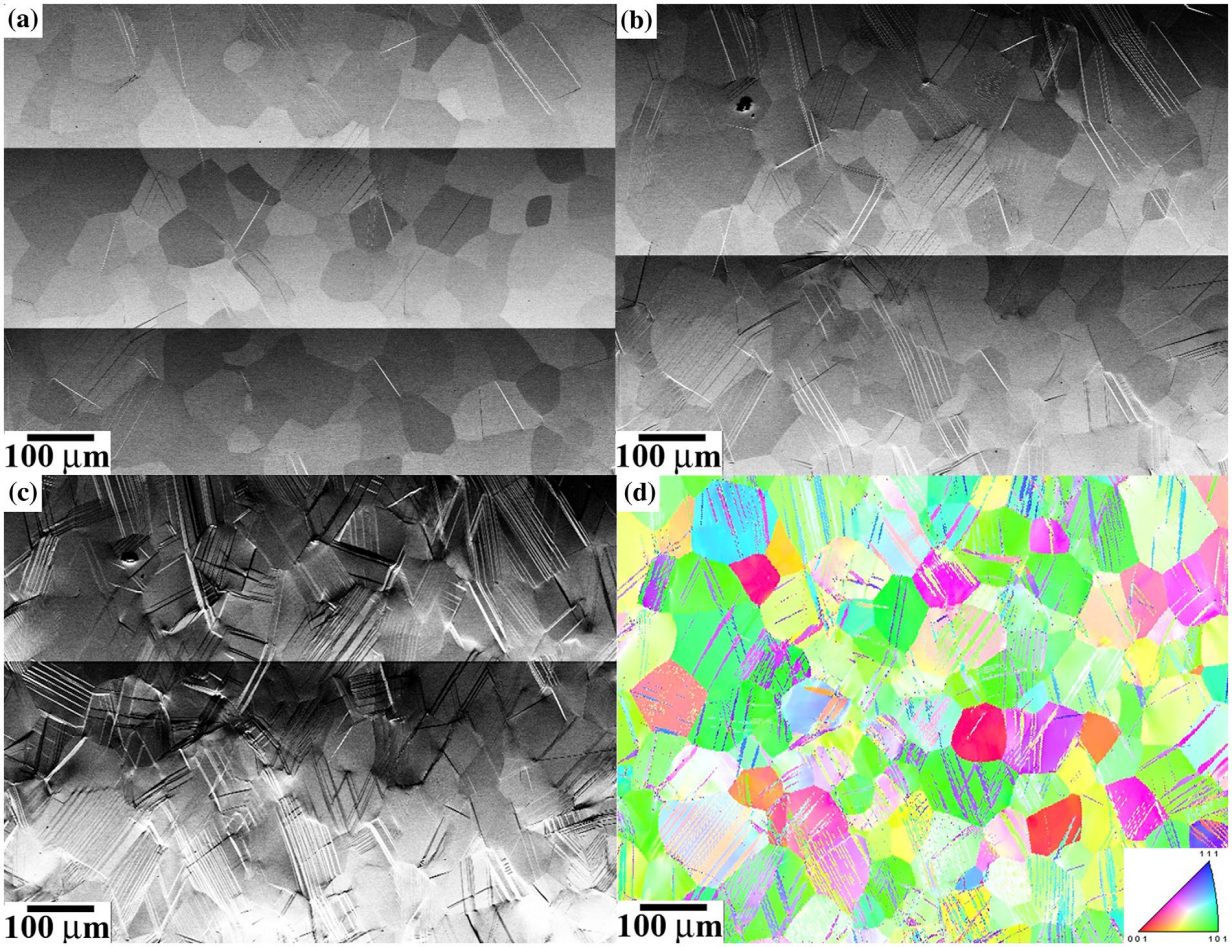
BSE-SEM images of the evolution of {332}〈113〉 twin structure upon deformation in the LG sample. (a) 380 MPa/*ε*: 0.3%; (b) 430 MPa/*ε*: 0.9%; (c) 530 MPa/*ε*: 8.8%. (d) EBSD map along tensile direction. Sample deformed to 530 MPa/*ε*: 8.8%.

Figures 4 and 5 show the evolution of {332}〈113〉 twin structure upon *in situ* SEM testing in a microstructural region in the center of the SG and LG samples. BSE-SEM images of Figures 4 and 5 correspond to overview examples of large surface areas and were not used in the statistical analysis of twinning. Specifically, Figure 4 shows BSE-SEM images of the twin structure in the SG sample tensile deformed to 425 MPa/*ε*: 0.3% (a), 495 MPa/*ε*: 1.4% (b), and 560 MPa/*ε*: 8.1% (c). The *in situ* SEM tests reveal that twinning is activated readily after yielding in the SG sample, Figure 4(a). Upon straining, the number of twinned grains and twins per grain increase steadily. Most of the grains are twinned at *ε* ~ 3%. Figure 4(d) shows the inverse pole figure EBSD map along the tensile direction of the microstructure area shown in Figures 4(a)–(c) strained to 495 MPa/*ε*: 1.4%. This map reveals strong influence of crystallographic grain orientation on twin activity, as shown by the statistical analysis of Section 3.3. Figure 5 shows BSE-SEM images of the twin structure in the LG sample tensile deformed to 380 MPa/*ε*: 0.3% (a), 430 MPa/*ε*: 0.9% (b), and 530 MPa/*ε*: 8.8% (c). As in the SG sample, the *in situ* SEM tests also reveal that twinning is activated readily after yielding. Upon straining, the number of twins per grain increases in a higher rate than that in the SG sample. Details of the influence of grain size on twinning are provided in Section 3.4. The crystallographic analysis of twin variants from EBSD maps and twin plane trace analysis of BSE-SEM images reveals that the twin structure consists of primary twins (twin variants with the highest Schmid factor, i.e. *v*(1)) and secondary twins (*v*(2)–*v*(12)). Primary twins are seen to nucleate at grain boundaries (including triple lines and quadruple points) whereas secondary twins are nucleated at both grain boundaries and primary twin interfaces.

### 3.2. Apparent twinning stress

**Figure 6.**
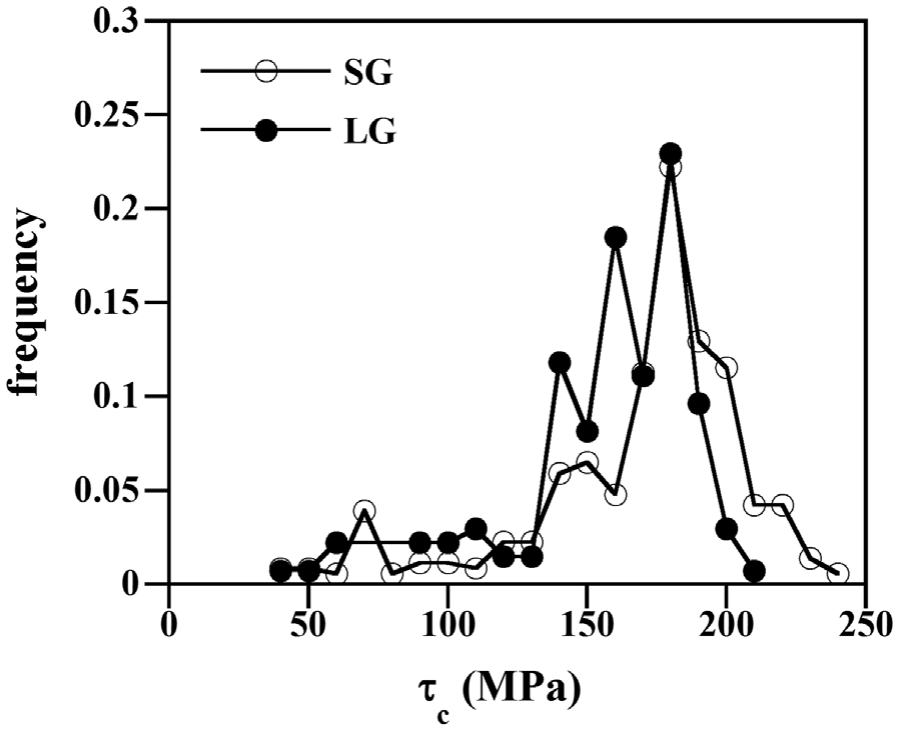
Frequency of the apparent critical resolved shear stress *τ*_*c*_ at which primary twins are nucleated and propagated in the SG an LG samples.

Notes: SG: Fine-grained sample; LG: Coarse-grained sample.

In order to evaluate the role of macroscopic applied stress on the nucleation and propagation behavior of {332}〈113〉 twins in the present β-Ti-15Mo alloy, we have estimated an apparent critical resolved shear stress *τ*_*c*_ at which primary twins occur. We have only evaluated the early stages of deformation (*ε* < 1.5–2.0%) where the number of twins per grain *n*_*tw*_ is small in order to exclude local stress effects from neighboring propagating twins. At this strain range, *n*_*tw*_ is smaller than 4–7, Figure 10(a). About 350 surface twinning events were investigated per sample by *in situ* SEM tensile testing at six strain levels ranging from the elastic regime to plastic strain of *ε* ~ 1.5–2.0%. We have only considered those twins that are nucleated at grain boundaries followed by observable propagation in the grain interior, typically until the nearest grain boundary. *τ*_*c*_ was determined as *τ*_*c*_ = *m*·*σ* where m is the Schmid factor of the activated twin variant and *σ* the macroscopic stress at which the twin was detected. *τ*_*c*_ can be considered as an apparent critical stress for twin nucleation and propagation since only considers the applied macroscopic stress resolved on the twin system. The role of local stress fields from neighboring twins and twin back-stresses, and intergranular compatibility stresses on twinning stress are not considered in the present approach. These effects are currently analyzed by crystal plasticity approaches [33–35]. Therefore, the resulting apparent critical shear stresses will overestimate the real critical shear stress in some grains and underestimate in others [31,36]. In order to minimize these effects and obtain a reasonable estimation of the critical resolved shear stress by surface analysis, we have performed a large statistical analysis of surface twinning events [31]. We consider that the median value of the frequency distribution of the apparent stress obtained by surface analysis provides a reasonable estimation of the critical resolved shear stress for twinning. Figure 6 shows the frequency distribution of *τ*_*c*_ for both samples, i.e. SG and LG. This plot indicates that most of the primary twins (85% of the observed twins) in the present β-Ti-15Mo alloy occur at *τ*_*c*_ = 140–220 ΜPa. Some twins are propagated at very low stress, i.e. *τ*_*c*_ < 100 MPa, which corresponds to the macroscopic elastic regime. These twins are likely associated to nucleation events occurring at specific microstructural features containing stress concentrators such as triple points. Figure 6 also shows that average grain size has significant influence on the stress range of *τ*_*c*_. The plot reveals that in the grain size range investigated here (40–120 μm), the median value of the distribution of *τ*_*c*_ of the coarse-grain size sample is shifted toward higher stress levels. Specifically, the median value of the stress distribution is *τ*_*c*_ = 180 ± 20 MPa and *τ*_*c*_ = 165 ± 25 MPa for SG and LG, respectively.

### 3.3. Crystallographic grain orientation effects on twinning

**Figure 7.**
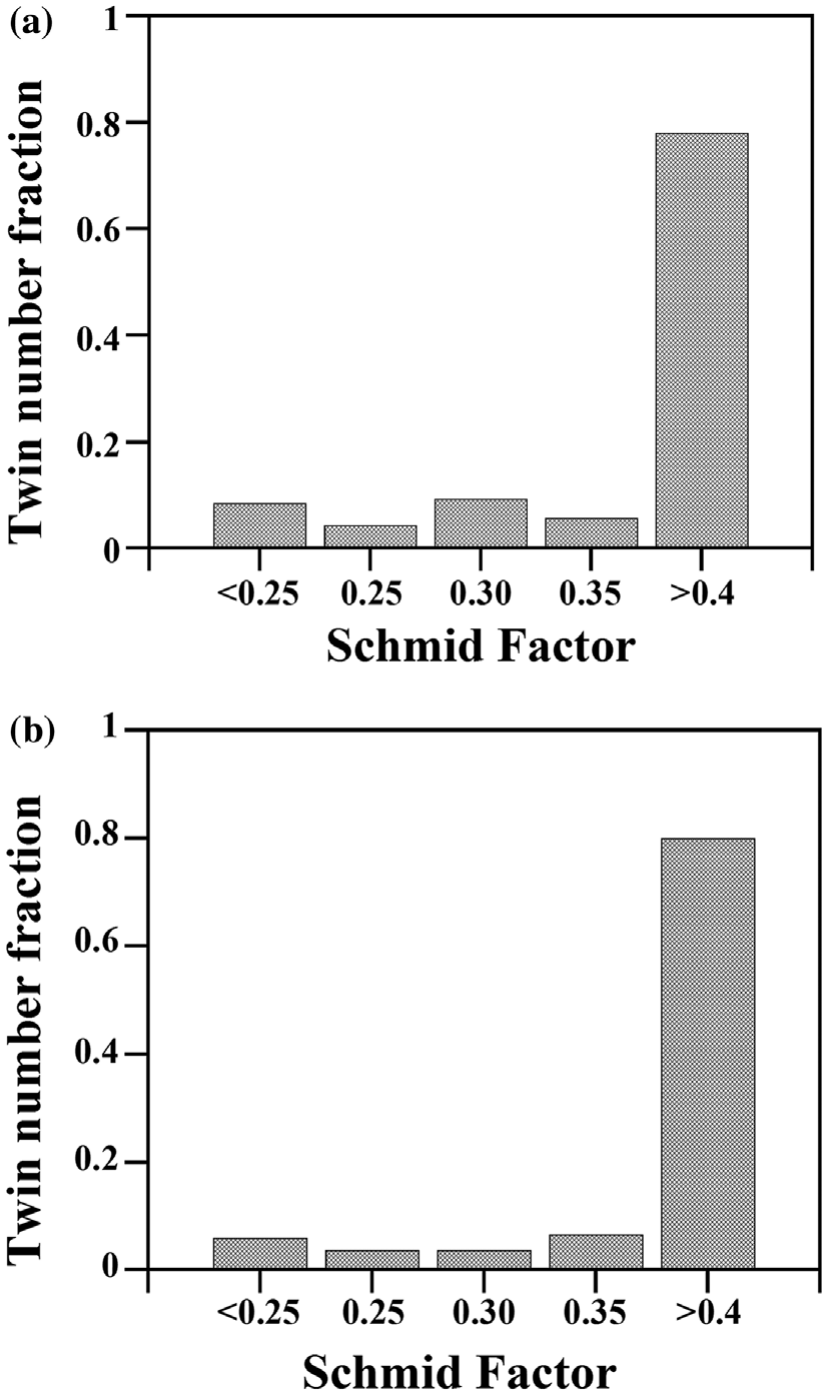
Twin number fraction as a function of the highest Schmid factor for twinning *m*(1) of the active twin variants in the SG (a) and LG sample (b), respectively.

**Figure 8.**
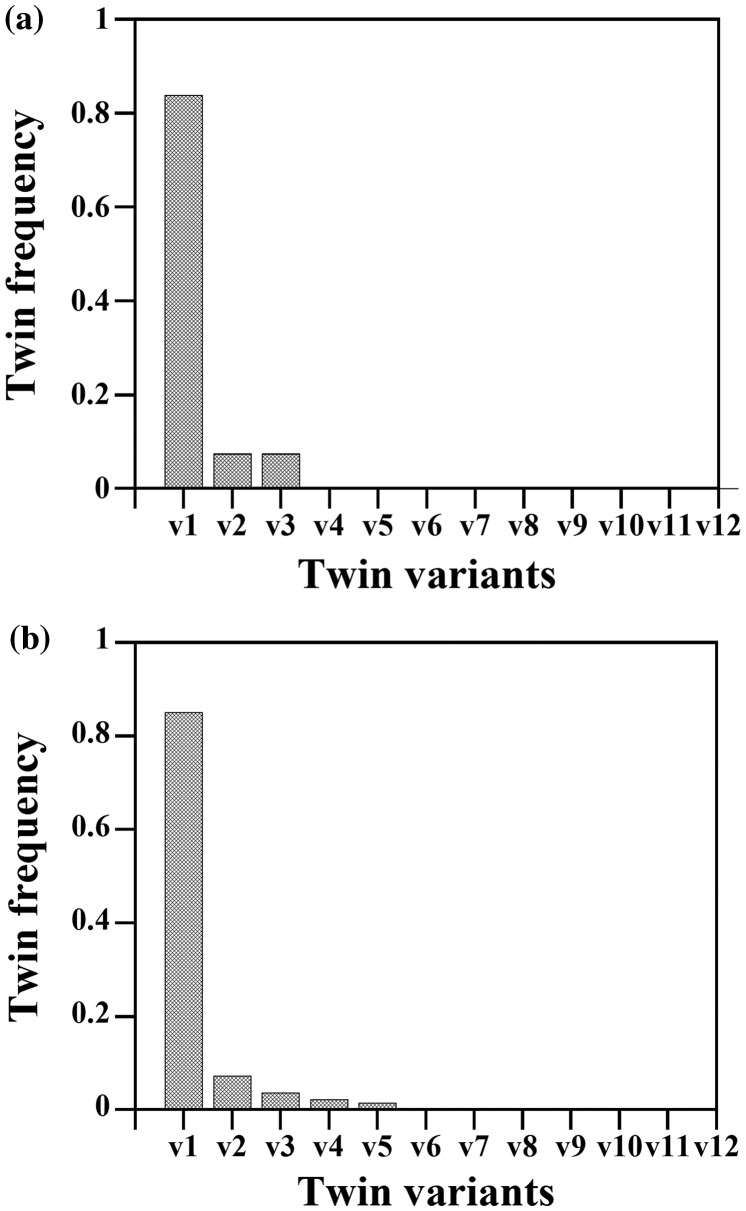
Twin frequency of activated twin variants (*v*(1) … *v*(12)) in the SG (a) and LG samples (b), respectively.

Figure 7 shows the distribution of the highest Schmid factor for twinning of the active twin variants in the SG (a) and LG samples (b), respectively. This plot reveals that most of the active twin variants follow Schmid law with respect to the macroscopic applied stress, namely, 72.4% of the twinned grains in the SG sample and 80.0% of the twinned grains in the LG sample are favorably oriented for twinning, i.e. *m* > 0.4. Interestingly, this plot also shows that twins appeared in grains over a wide range of *m*, i.e. they also appeared in grains oriented less favorably for twinning. The relative frequency of the twin variants in the SG and LG samples is analyzed in Figure 8. This plot shows that most of the active twin variants are *v*(1) type, namely, 83.5% in the SG sample and 85.2% in the LG sample. The probability of nucleating twin variants with low geometric Schmid factor (*v*(2)–*v*(12)) is low (7.5–9.0%).

### 3.4. Grain size effects on twinning

**Figure 9.**
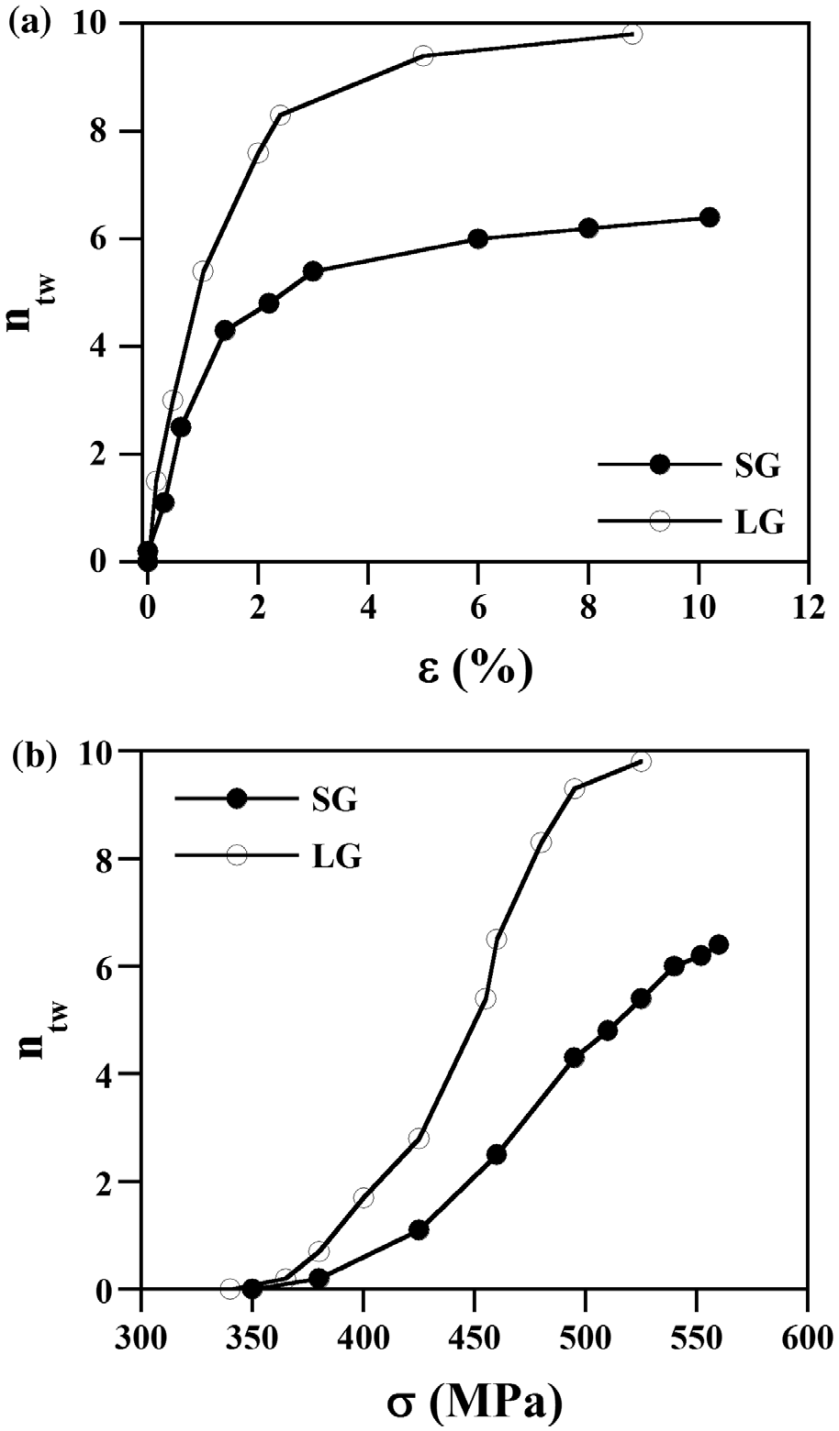
Evolution of the number of twins *n*_*tw*_ with plastic strain, *ε*, (a) and macroscopic stress, *σ*, (b).

Notes: SG: Fine-grained sample; LG: Coarse-grained sample.

We have evaluated the role of grain size on twinning by the analysis of the evolution of the number of primary twins upon straining. Figure 9 shows the number of twins per grain *n*_*tw*_ as a function of the plastic strain (a) and macroscopic stress (b). These plots show that the evolution of *n*_*tw*_ with plastic strain follows the same trend on both samples. Specifically, *n*_*tw*_ gradually increases with plastic strain up to strain level of *ε* ~ 1.5–2.5% (*n*_*tw*_ ~ 4.5 in SG; *n*_*tw*_ = 8.3 in LG). Thereafter, it rises in a moderate fashion. This plot also reveals that *n*_*tw*_ scales with grain size, i.e. *ntw* in LG is about 1.2–1.5 times higher than that in SG. The evolution of *n*_*tw*_ with stress exhibits a more complex trend, which is related to the flow stress dependence on several twin parameters such as twin thickness and twin spacing [37,38]. Figure 9(b) shows that in the SG sample *n*_*tw*_ scales almost linearly with stress within the whole deformation range, up to *σ* ~ 540 MPa. On the other hand, in the LG sample *n*_*tw*_ scales linearly with stress only within the early stages of deformation, up to *σ* ~ 450 MPa. From that point on, *n*_*tw*_ increases remarkably (from 4.0 to 9.8) in a small stress range of about 75 MPa (from 450 to 525 MPa). The flow stress scales with twin spacing, which is determined by the twin thickness and twin area fraction [38]. At the same value of *n*_*tw*_, the SG sample contains a twin area fraction about 3 times higher than that of the LG sample (assuming same twin thickness and homogeneous twin distribution). Accordingly, the influence of *n*_*tw*_ on the flow stress in the SG sample is higher than that in the LG sample, as Figure 9(b) shows.

**Figure 10.**
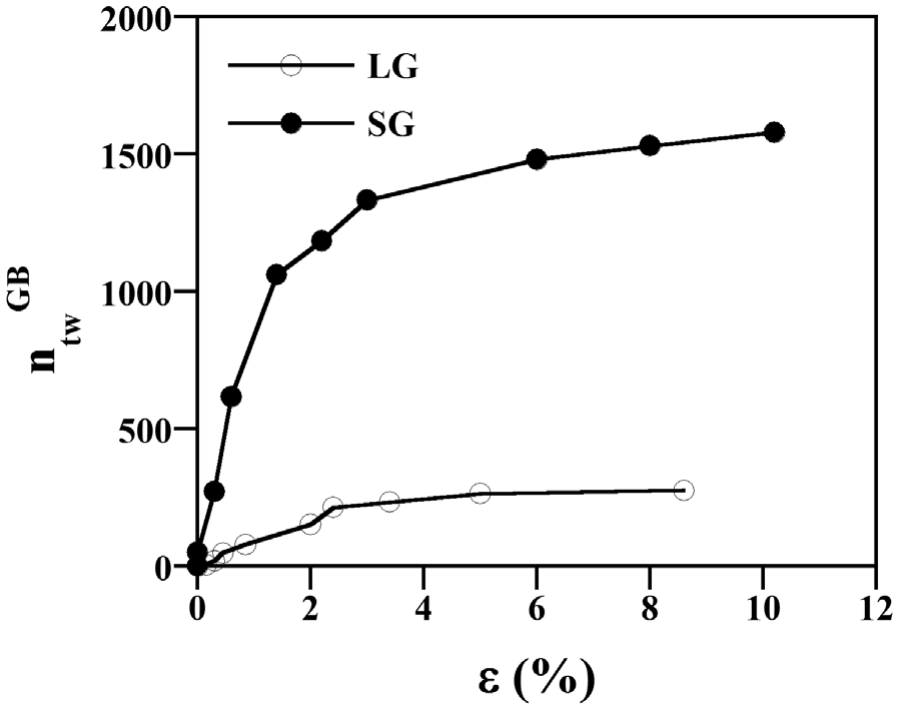
Evolution of the number of twins per grain boundary area $n_{\mathrm{tw}}^{\mathrm{GB}}$with plastic strain, *ε*.

Notes: SG: Fine-grained sample; LG: Coarse-grained sample.

It is also worth to analyze the effect of grain size on the number of twins per unit of grain boundary area, $n_{\mathrm{tw}}^{\mathrm{GB}}$. $n_{\mathrm{tw}}^{\mathrm{GB}}$ is defined as:

(1) $$n_{\mathrm{tw}}^{\mathrm{GB}}\;=n_{v}/\left(4/D\right)$$

where *n*_*v*_ is the number of twins per unit volume and *D* is the grain size. If we consider twins as oblate spheroids, then *n*_*v*_ is related to the number of twins per unit area, *n*_*a*_, as follows [39]:

(2) $$n_{v}=n_{a}/l\;k\left(q\right)$$

where *l* is the twin length and *k*(*q*) is a geometrical function of the twin aspect ratio. According to our observations, *l* can be set as *D* and *k*(*q*) = 0.8–0.9. *n*_*a*_ was determined from SEM images. The evolution of $n_{\mathrm{tw}}^{\mathrm{GB}}$with strain level for both samples is plotted in Figure 10. This figure reveals that $n_{\mathrm{tw}}^{\mathrm{GB}}$ is significantly reduced with grain size (about six times). Similar effect has been reported in Mg [24]. It can be also seen that both samples exhibit similar evolution of $n_{\mathrm{tw}}^{\mathrm{GB}}$ with strain, namely, a rapid increase of $n_{\mathrm{tw}}^{\mathrm{GB}}$ with plastic strain up to strain level of *ε* ~ 2.5–3.0%, and thereafter the increase is slightly moderated.

## 4. Discussion

### 4.1. Apparent twinning stress

The nucleation and propagation of primary {332}〈113〉 twins in the present β-Ti-15Mo alloy occurs over a specific apparent critical resolved shear stress range, i.e. *τ*_*c*_ = 140–220 MPa. This finding agrees with former reports in single crystals of β-Ti alloys. These works have reported a large scatter in the critical resolved shear stress (CRSS) *τ*_*cr*_ for {332}〈113〉 twinning. Specifically, stress ranges of Δ*τ*_*cr*_ ~ 140 MPa in a Ti-15Mo-5Zr (wt.%) alloy [40], Δ*τ*_*cr*_ ~ 150 MPa in a Ti-22%V (wt.%) alloy [5], and Δ*τ*_*cr*_ ~ 50 MPa in a Ti-24%V (wt.%) alloy [5] have been reported. Interestingly, these stress ranges are similar to that found in the present study, i.e. Δ*τ*_*c*_ ~ 80 MPa. These findings suggest that {332}〈113〉 twinning in β-Ti alloys occurs over a certain critical resolved stress range. This effect can be explained as follows. From a classical mechanical standpoint, a constant critical stress can be assigned to deformation twinning, in a similar fashion than that in dislocation slip [1]. However, if we consider the presence of varying stress concentrators associated to microstructural (i.e. triple lines) and macroscopic features (i.e. sample surface roughness), together with intergranular compatibility stresses it yields to a variable twinning stress. Recent micromechanical models on twin nucleation in hcp materials that take into account the grain boundary defect structure have also demonstrated that twinning stress is not a constant parameter [41,42]. These approaches consider that the stress field at a grain boundary contains short-range local fluctuations resulting in a variable twin nucleation stress and hence, in a variable critical resolved shear stress for twinning.

### 4.2. Crystallographic grain orientation effects on twinning

The present statistical analysis of the influence of crystallographic grain orientation on primary {332}〈113〉 twinning shows that most of the twins follow Schmid’s law with respect to the macroscopic stress. Twins were formed in the most favorable orientations in 72.4 and 80% of the twinned grains in the SG and LG samples, respectively. These observations agree with several reports on {332}〈113〉 twinning in metastable β-Ti alloys [7,13,14]. Interestingly, our study shows that twinning also occurs in grains with unfavorable orientations (20.0–27.6% of the investigated grains). Similar observations have been recently reported on {10–12} twinning in Mg and Zr alloys [16,17,20,43,44]. However, the fraction of twinned grains with unfavorable orientations observed in these alloys (~40 to 60%) is much higher than that observed in the present study. The growth of twin variants with low macroscopic Schmid factor has been associated to several phenomena such as local strain accommodation effects [17,45], competition between twin back-stress and in-grain stress state [15,33,46,47], and stress fluctuations at grain boundaries [41,42]. Further work is required to determine the underlying mechanism of the growth of {332}〈113〉 twin variants with low macroscopic Schmid factor in β-Ti alloys.

Interestingly, our analysis also reveals that most of the growth twins correspond to the higher stressed variant (83.5% of the twins in the SG sample and 85.2% of the twins in the LG sample). This result agrees with that obtained by Hanada et al. [5] in single crystals of β-TiNb alloys. Beyerlein et al. [41,42] have shown that twin nucleation and twin growth are governed by different stress states. Twin nucleation and, hence twin variant selection, are governed by the localized fluctuating stress state at grain boundaries produced by features such as dislocation pile-ups and impinging twins from neighboring grains. On the other hand, twin growth is controlled by the competition between the twin back-stress field and the in-grain stress field [15,46,47]. Our data suggest that in the present β-Ti-15Mo alloy, the twin systems with the highest Schmid factor are also those systems that are most strongly stressed at the grain level. Accordingly, twin growth is favored on the twin systems with the highest geometric Schmid factor. This finding suggests that the role of local stress fields on primary twinning in the present β-Ti-15Mo alloy is limited.

### 4.3. Grain size effects on twinning

We have investigated grain size effects on twinning by the analysis of the evolution of the number of twins per grain *n*_*tw*_ and the number of twins per grain boundary area $n_{\mathrm{tw}}^{\mathrm{GB}}$ with strain. As expected, the present study reveals that *n*_*tw*_ scales with grain size, namely, *n*_*tw*_ in LG is about 1.2–1.5 times higher than that in SG. On the other hand, $n_{\mathrm{tw}}^{\mathrm{GB}}$ significantly decreases with grain size. In particular, in the present average grain size range (40–120 μm), $n_{\mathrm{tw}}^{\mathrm{GB}}$ decreases about 6 times at a given strain level. The strong influence of grain size on *n*_*tw*_ and $n_{\mathrm{tw}}^{\mathrm{GB}}$ has been also reported on different twinning systems in hcp metals such as Mg, Zr and Ti [15,16,24]. The scaling of *n*_*tw*_ with grain size has been recently explained by a probabilistic twin nucleation model where twin nucleation is described as a dissociation process of grain boundary defects into twinning partials to create a twin nucleus [41,42]. The number of successful conversion events is considered to follow a stochastic process where the rate is assumed to increase with local stress. On the other hand, the scaling of $n_{\mathrm{tw}}^{\mathrm{GB}}$ with grain size can be attributed to the lower resolved stress acting on a twin system in the coarse-grain sample. The plot of the frequency of the apparent critical resolved shear stress *τ*_*c*_, reveals a grain size effect on *τ*_*c*_. Specifically, the median value of the stress distribution is *τ*_*c*_ = 180 ± 20 MPa and *τ*_*c*_ = 165 ± 25 MPa for SG and LG, respectively. We can expect that if *τ*_*c*_ exhibits grain size dependence, the average resolved stress on the active twin system exhibits grain size dependence as well. This finding agrees with that observed by Ghaderi and Barnett in Ti and a AZ31Mg alloy [24]. In particular, these authors have suggested the following empirical relation between $n_{\mathrm{tw}}^{\mathrm{GB}}$ and *τ*, $n_{\mathrm{tw}}^{\mathrm{GB}}$ ~ *τ* ^2^.

## 5. Conclusions

We have investigated microstructure-twinning relations in the {332}〈113〉 twin system by quantitative characterization of deformation twin structure in a polycrystalline β-Ti-15Mo (wt.%) alloy by *in situ* SEM and EBSD. Statistical analysis of the evolving surface twin structure upon deformation to *ε* ~ 10% was performed to identify correlations among several microstructural features and twin characteristics. The following conclusions can be drawn:

• Our estimation of an apparent critical resolved shear stress *τ*_*c*_ at which primary twinning occurs reveals that twinning in the grain size range studied here (40–120 μm) occurs at a specific applied stress range, i.e. *τ*_*c*_ = 140–220 MPa. We observe that *τ*_*c*_ exhibits a grain size dependence.
• At the early stages of deformation (*ε* < 1.5–2.0%), most of the primary twins (~70–80% of the analyzed twins) follow Schmid’s law with respect to the macroscopic stress. We also observe that most of the growth twins (~85% of the analyzed twins) correspond to the higher stressed variant. These findings suggest that the twin systems with the highest Schmid factor are also those systems that are most strongly stressed at the grain level.
• In the grain size range studied here (40–120 μm), the number of twins per grain *n*_*tw*_ and number of twins per grain boundary area $n_{\mathrm{tw}}^{\mathrm{GB}}$ exhibit strong grain size dependence. We ascribe these effects to the grain size dependence of twin nucleation stress and apparent critical resolved shear stress *τ*_*c*_, respectively.

## Disclosure statement

No potential conflict of interest was reported by the authors.
